# Social engagement and physical frailty in later life: does marital status matter?

**DOI:** 10.1186/s12877-021-02194-x

**Published:** 2021-04-15

**Authors:** Yi Wang, Zhuo Chen, Chengchao Zhou

**Affiliations:** 1 Centre for Health Management and Policy Research, School of Public Health, Cheeloo College of Medicine, Shandong University, Jinan, 250012 China; 2 NHC Key Laboratory of Health Economics and Policy Research, Shandong University, 44 Wen-hua-xi Road, Jinan, 250012 Shandong China; 3 College of Public Health, University of Georgia, Athens, GA 30602 USA; 4 School of Economics, University of Nottingham Ningbo China, Ningbo, 315100 China

**Keywords:** Social engagement, Marital status, Frailty, Fixed-effects model, China health and retirement longitudinal study

## Abstract

**Background:**

Physical frailty is a common characteristic of older people with the ageing process and has been viewed as a major public health issue. The longitudinal association between different social engagement and physical frailty among older people has not been explored adequately in China. Marital status forms a critical context for the link between social engagement and frailty among older people, which might constitute a moderating process. The purpose of the present study is to investigate the longitudinal association between social engagement and the changes in physical frailty among Chinese older adults, and to examine whether the association between social engagement and frailty differs by marital status.

**Methods:**

The data use in this study were from the data from the China Health and Retirement Longitudinal Study aged 60+ years from 2011 to 2015. A total of 6575 respondents who participated in at least one follow-up wave were included in the analysis. The relationship between social engagement and changes in frailty over time, and the moderating role of marital status were estimated using individual fixed-effects models. Sensitive analyses were conducted to test the robustness of the results.

**Results:**

After adjusting the confounders, participants who interact with friends (Coef: -1.309, *P* < 0.001), engaging in hobby groups (Coef: -1.189, *P* < 0.001), engaging in sports groups (Coef: -0.945, *P* = 0.001), and volunteering (Coef: -1.957, *P* = 0.001) with a frequency of almost daily had a significantly lower frailty risk than participants who never engaging in those activities. The association between frequent engaging in hobby groups and physical frailty was strongest for unmarried than married older adults (Coef: -1.325, *P* = 0.031).

**Conclusions:**

Frequent social engagement might help to decrease the risk of frailty in the Chinese older population. This finding has important implications for public health policy and encourages the incorporation of a broad range of social engagement into the daily lives of older individuals. Specially, encouraging unmarried older adults to engage in intellectual activities, such as playing chess or Mahjong with others, may be an effective way to reduce physical frailty.

**Supplementary Information:**

The online version contains supplementary material available at 10.1186/s12877-021-02194-x.

## Background

The population is ageing worldwide. Population ageing presents tremendous challenges and has far-reaching implications for individuals, families, and communities. One of the most profound challenges is the increasing multiple adverse health conditions as people age. The term *frailty* is used to characterize the evolving health conditions during the ageing process. It is defined as a multifactorial syndrome that reduces one’s physiologic reserve [1] and leads to a state of increased vulnerability to adverse health outcomes, such as falls, fractures, disability, hospitalization, as well as an overall negative state of health [2–4]. A recent study also shows physical frailty is associated with a higher risk of developing severe disease among older COVID-19 patients [5]. Physical frailty also reflects an accumulation of health deficits during an individual’s life, and the Frailty Index (FI) is one of the most common instruments to measure [6, 7]. Frailty not only impacts older individuals but also challenges societies and health care systems. Previous studies have also demonstrated that frailty could increase healthcare cost [8]. Therefore, frailty is viewed as one of the major public health challenges in rapidly ageing societies [9].

Physical frailty is a reversible process when effective interventions are employed [10]. Most interventions are clinic based, target those who are already frail or pre-frail [11], including cognitive training [12], nutritional support [13], comprehensive geriatric assessment [14], exercise [15], or their combinations [16]. In recent years, increasing studies have begun to explore the association between social factors and frailty using population-based samples [17, 18]. Among the social determinants of health, social engagement emerges as an important concept for building and maintaining relationships among older people. Previous studies and theories on social engagement provide substantial theoretical motivation for understanding the link between social engagement and frailty in later life [19]. Social engagement, as a core component of “*Active Ageing*” proposed by the World Health Organization [20], is defined as “a person’s involvement in activities that provide interaction with others in society or the community in order to enhance the quality of life as people age” [20]. Social engagement takes many forms, including interaction with friends, volunteering, and engaging in hobby groups [21], and is considered as one of the objective dimensions of social isolation [22]. One study shows that social engagement is associated with physical frailty using cross-sectional data in Hong Kong [23]. Nevertheless, frailty status and social engagement changes over time, and the longitudinal association between different types of social engagement and physical frailty among older people has not been explored adequately in China.

Marital status forms a critical context for the link between social engagement and frailty among older people, which would constitute a moderating process. Many studies have shown that married people are healthier than unmarried people in later life. For example, married people tend to have lower risk of mortality [24] and suffer from fewer chronic conditions [25]. Theoretically, being married is associated with better social engagement, social support, and social integration [26]—all factors linked to better health and well-being [27], including less physical frailty. A recent systematic review [17] pooled 35 cross-sectional studies and finds being unmarried is associated with increased frailty. Three longitudinal studies also confirm the same results from Italy [28, 29] and the United States [30]. While the association between marriage and frailty has been found, the role of marital status as a moderator in the relationship between social engagement and frailty remains unknown. Previous studies have shown that marital status plays a moderating role in the relationship between some factors and health in later life. For example, Gyasi [31] finds that the association between self-rated health and functional decline among older people are modified by marital status. Kail [32] also finds that marital status moderates the relationship between depressive symptoms and chronic conditions on subsequent functional limitations among older people. However, there is no empirical research on the moderating role of marital status between social engagement and frailty. Based on the review of previous studies, it is necessary to investigate the moderating role of marital status in the association between social engagement and frailty in later life. Specifying the role of marital status in the link between social engagement and frailty will help policy makers to design more effective and precise intervention strategies on frailty prevention.

The purpose of the present study is 2-fold. First, while social engagement has been found to be associated with positive physical and mental health among older people, whether this association would extend to physical frailty is still unclear, especially lacking the longitudinal evidence in mainland China. Thus, the first aim is to investigate the longitudinal association between social engagement and the changes in physical frailty among Chinese older adults. Second, marital status forms a critical context for the link between social engagement and frailty among older people, and married people are healthier than unmarried people in later life, thus the second aim is to examine whether the association between social engagement and frailty differes by marital status. To achieve the two aims, we use data from the China Health and Retirement Longitudinal Study (CHARLS)**,** which is a nationwide survey aiming to provide sufficient micro-level longitudinal data for aging studies. Its design is similar to that of the Health and Retirement Study (HRS) in the United States, the English Longitudinal Study of Aging (ELSA), and the Survey of Health, Aging and Retirement in Europe (SHARE). We hypothesize that: (a) Four types of social engagement, i.e., interaction with friends, engaging in hobby groups, sports groups, and volunteering, are all associated with the reduced frailty among Chinese older people; (b) The association between social engagement and frailty is moderated by marital status.

## Methods

### Study design and data sources

We used panel data from the three waves of the China Health and Retirement Longitudinal Study (CHARLS) in 2011, 2013, and 2015. The CHARLS collected high-quality data via in-person interviews with a structured questionnaire, and adopted a four-stage, stratified, cluster sampling method to enroll community-dwelling residents from 450 villages and 150 counties in 28 provinces in China. Details of its sampling technique have been reported elsewhere [33]. With the response rate of 80.5%, the total sample size of the CHARLS baseline survey in 2011 was 17,714 respondents aged 45 years or older, who were followed up once every 2 years. For the current study, we included the respondents who were aged 60 years and older in order to better focus on the frailty of older adults, and there were 7655 respondents aged 60 and older at baseline. Then, we excluded 386 respondents who had memory-related diseases to reduce recall biases. In wave 2, 996 respondents were lost to follow up with the rate of 13.70%, and the lost-to-follow up rate was 10.17% in wave 3. A total of 6575 respondents who participated in at least one follow-up wave were included in the analysis.

### Physical frailty

In this study, physical frailty was defined as the accumulation of health deficits during an individual’s later life, and we used the Frailty Index (FI) to estimate the frailty of each respondent. FI can provide a more comprehensive picture of older adults’ overall health, and is particularly useful for predicting adverse health outcomes [34, 35]. Although studies using frailty index often do not include the same number or type of indicators to estimate frailty, it is demonstrated that the results are consistent as long as the major domains of health are included in the index (i.e., ADLs, IADLs, chronic diseases, psychological characteristics, and cognitive function) [36]. In this study, the FI was computed following a standard procedure (such as associated with health, increased with age, not saturate too early, and cover a range of systems) [7, 36–38], and the items covered all the major health domains, including self-rated health, activities of daily living (ADL), instrumental activities of daily living (IADL), cognitive function, chronic diseases, and psychological characteristics (see Supplementary Table 1). Each item was dichotomous or ordinal, measured on a scale of 0 to 1 to represent the severity of health deficits, and then each of the deficit points was summed and divided by the total number of deficits evaluated and then multiplied by 100. Therefore, we obtained a FI score with a theoretical range from 0 (no deficits present) to 100 (all deficits present). For individuals with missing data on some deficits, we excluded the deficits with missing information from both the denominator and the numerator. If more than 20% of the deficits were missing, the FI was treated as missing. This is a commonly used criterion, allowing for maximum use of available data [39]. A higher FI score indicated more health problems and hence more frail.

### Social engagement

Social engagement was defined as involvement in any type of social activity during the study period. Respondents were asked how often they took part in the following four types of activities in the last month: (1) interaction with friends; (2) hobby groups (participated in chess, Mahjong, card game with others or community club, etc.); (3) sports groups (went to a sporting event, such as square dance, Tai Chi or other kinds of sports club, etc.); (4) voluntary work (participating in voluntary or charity work or providing help to others without financial compensation). For each activity, an additional question was asked about the frequency of engagement, which was categorized as: often (almost daily), some of the time (almost every week), not regularly (about once a month), and never.

### Marital status

Marital status was divided into two categories, i.e., married and unmarried status (cohabiting, separated/divorced, widowed, or never married). Cohabiters were included in the unmarried groups, because they might not receive the same levels of socio-psychological and economic benefits as married individuals due to a lack of institutional legitimacy and less commitment based on previous studies [27].

### Control variables

Potential confounding variables in this study included age, financial situation (household income per capita), currently working (yes or no), smoking status, alcohol consumption, multi-morbidity (co-existing of two or more chronic diseases), self-perceived health status, depression, physical activity, and wave. We did not control for time-independent factors, such as sex, education, baseline age, etc., because fixed effects regression models only use within-person variation, and time-invariant factors will be absorbed in the fixed effects. We measured depression using the 10-item Center for Epidemiologic Studies Depression Scale (CES-D), and participants with CESD-10 scores above 10 were classified as having depressive symptoms [40]. Physical activity was measured by asking whether the respondents did any vigorous, moderately energetic or mildly energetic physical activity for at least 10 min every week. However, according to the official statement from the CHARLS team, the questions on physical activity were asked to a random subsample of half the sample. Consequently, in order to preserve the statistical power, we only controlled physical activity in the sensitivity analyses using multiple imputation technique to test the robustness of the results. Wave was included to capture any individual-level idiosyncratic disturbances over time.

### Analytical strategy

We first examined the descriptive characteristics of the study sample separated by wave. Then, longitudinal linear fixed-effects (FE) models were used to examine the association between changes in social engagement and changes in FI within each older adult. Applying a fixed effects model on panel data reduces potential bias due to unobserved heterogeneity [41]. In models 1 to 5, each type of social engagement and marital status were included separately, whereas, in model 6, we included all at once. All models were adjusted for confounding variables. Furthermore, we included interaction terms between the four types of social engagement and marital status to explore whether the associations differed by marital status in models 7 to 10. The FE model equation was:

1 $$ {FI}_{it}={\mathrm{Z}}_i\alpha +{\mathrm{X}}_{it}{\upbeta}_1+{M}_{it}{\upbeta}_2+{X}_{it}^{\prime }{\upbeta}_3+{U}_t+{\upvarepsilon}_{it} $$

*FI*_*it*_ denotes the frailty index for individual *i* at wave *t*. Z_*i*_ refers to the fixed effects, which includes all observed and unobserved time-invariant covariates. X_*it*_ and *M*_*it*_ indicate four types of social engagement and marital status for individual *i* at wave *t*, respectively. ${X}_{it}^{\prime }$ indicates time-varying control variables. *U*_*t*_ denotes wave effects, and *Ɛ*_*it*_ is the error term.

To test the feasibility and robustness of the FE model, we conducted several sensitivity analyses. First, FE models may yield biased and inconsistent results when “strict exogeneity” cannot be assumed. To minimize the potential impact of reverse causality, we conducted a test for the endogenous selection (i.e., reverse causality) by using the following equation [42]:

2 $$ \mathrm{Social}\_{\mathrm{engagement}}_t={a}_1+{b}_1\ast {FI}_{t-1}+{c}_1\ast \left({\sum}_{t-1=1}^{n=3}{FI}_{\mathrm{t}-1}\right) $$

We tested whether social engagement (t) can be predicted by the previous wave’s frailty (*FI*_*t-1*_) net of the proxy for the fixed effect $\left({\sum}_{t-1=1}^{n=3}{FI}_{t-1}\right)$. If the coefficient b is not significant, then the reverse causality is unlikely. This approach has been shown to be effective in previous studies [43] (see Supplementary Table 2).

Second, F-test was used to compare the pooled ordinary least squares (POLS) and FE model. We then used Hausman-test between the FE model and the random-effects (RE) model. Next, we investigated whether it was necessary to control for time-fixed effects in the models. Finally, to test the robustness of the results, we used multiple imputation techniques to reduce the potential biases resulting from missing data in the frailty index and control variables. Multiple imputation involves replacing missing values with predictions based on other observed variables by chained equation method [44]. All analyses were performed with Stata 14.2 (StataCorp, College Station, TX).

## Results

Table 1 shows the descriptive statistics separated by sample wave. Of the 6575 participants at baseline, the mean age was 68 years, 49.79% were men, and 44.78% with a primary school education. The majority of respondents were married (75.21%), currently working (53.16%), did not smoke (58.27%), consume alcohol (69.52%), with multi-morbidity (46.10%), and without depressive symptoms (62.05%). Regarding social engagement, the most popular type was interaction with friends (16.73% of the respondents interact with friends almost daily). The average FI score of the respondents was 18.65 at baseline. The following two waves saw the average FI score gradually increased.

**Table 1:** Sample characteristics of the respondents by wave

	Baseline (2011)	Wave 2 (2013)	Wave 3 (2015)
	*N* = 6575	*N* = 6273	*N* = 5836
Time-invariant characteristics			
Gender			
Men	3268 (49.70)	3123 (49.78)	2874 (49.25)
Women	3307 (50.30)	3150 (50.22)	2962 (50.75)
Educational attainment			
Illiterate	2420 (36.81)	2320 (36.98)	2145 (36.75)
Primary school	2944 (44.78)	2816 (44.89)	2639 (45.22)
Middle school or above	1211 (18.42)	1137 (18.13)	1052 (18.03)
Time-varying characteristics			
Age, mean (SD)	67.97 (6.67)	69.98 (6.66)	71.54 (6.40)
Marital Status			
Unmarried	1630 (24.79)	1671 (26.64)	1671 (28.63)
Married	4945 (75.21)	4599 (73.31)	4164 (71.35)
Missing	0 (0.00)	3 (0.05)	1 (0.02)
Financial situation, mean (SD)	7366.98 (13,482.04)	7600.20 (17,186.05)	6165.00 (28,279.95)
Currently working			
Yes	3495 (53.16)	3134 (49.96)	2763 (47.34)
No	3053 (46.43)	3060 (48.78)	3023 (51.80)
Missing	27 (0.41)	79 (1.26)	50 (0.86)
Smoking status			
Yes	2743 (41.72)	2893 (46.12)	2773 (47.52)
No	3831 (58.27)	3334 (53.15)	3046 (52.19)
Missing	1 (0.02)	46 (0.74)	50 (0.86)
Alcohol consumption			
Yes	2001 (30.43)	1871 (29.83)	1763 (30.21)
No	4571 (69.52)	4344 (69.25)	4052 (69.43)
Missing	3 (0.05)	58 (0.92)	21 (0.36)
Multi-morbidity			
No chronic condition	1596 (24.27)	1291 (20.58)	728 (12.47)
One chronic condition	1948 (29.63)	1732 (27.61)	1297 (22.22)
Multi-morbidity	3031 (46.10)	3250 (51.81)	3811 (65.30)
Depression			
Yes	2495 (37.95)	1791 (28.55)	1947 (33.36)
No	4080 (62.05)	4482 (71.45)	3889 (66.64)
Self-perceived health status			
Very good, good	1151 (17.51)	1384 (22.06)	1170 (20.05)
Fair, poor, or very poor	5424 (82.49)	4889 (77.94)	4666 (79.95)
Physical activity			
Yes	2307 (35.09)	1733 (27.63)	2292 (39.27)
No	331 (5.03)	292 (4.65)	391 (6.70)
Missing	3937 (59.88)	4248 (67.72)	3153 (54.03)
Social engagement			
Interaction with friends			
Never	4505 (68.52)	4125 (65.76)	4103 (70.31)
Not regularly	530 (8.06)	590 (9.41)	528 (9.05)
Almost every week	437 (6.65)	397 (6.33)	345 (5.91)
Almost daily	1100 (16.73)	1161 (18.51)	860 (14.74)
Missing	3 (0.05)	0 (0.00)	0 (0.00)
Hobby groups			
Never	5571 (84.73)	5262 (83.88)	4924 (84.37)
Not regularly	298 (4.53)	308 (4.91)	305 (5.23)
Almost every week	338 (5.14)	306 (4.88)	258 (4.42)
Almost daily	368 (5.60)	397 (6.33)	349 (5.98)
Sports groups			
Never	6154 (93.60)	5789 (92.28)	5482 (93.93)
Not regularly	57 (0.87)	57 (0.91)	52 (0.89)
Almost every week	66 (1.00)	61 (0.97)	39 (0.67)
Almost daily	298 (4.53)	366 (5.83)	263 (4.51)
Volunteer activities			
Never	6254 (95.12)	5661 (90.24)	5174 (88.66)
Not regularly	221 (3.36)	434 (6.92)	485 (8.31)
Almost every week	52 (0.79)	116 (1.85)	107 (1.83)
Almost daily	48 (0.73)	62 (0.99)	70 (1.20)
Frailty index, mean (SD)	18.65 (11.82)	20.06 (12.66)	23.05 (13.93)

The results of the FE regression are shown in Table 2. Age, financial situation, currently working, smoking status, alcohol consumption, multi-morbidity, self-perceived health status, depression, and wave were controlled in all specifications. The coefficients of taking each social engagement activity with a frequency of almost daily and married were all significantly negative in models 1 to 5. When all four social engagement and marital status were included in model 6, the coefficient of each type of social engagement with a frequency of almost daily remained statistically significant. To be specific, compared with none participation, interaction with friends (Coef: -1.309, *P* < 0.001), engaging in hobby groups (Coef: -1.189, *P* < 0.001), engaging in sports groups (Coef: -0.945, *P* = 0.001), and volunteering (Coef: -1.957, *P* = 0.001) almost daily were all significantly associated with reduced frailty. We also identified a similar association in not regular and some of the time groups, but the coefficients declines, such as interaction with friends (engagement not regularly: Coef: -0.568, *P* = 0.007; engagement some of the time: Coef: -1.152, *P* < 0.001) and hobby groups (engagement not regularly: Coef: -0.694, *P* = 0.011; engagement some of the time: Coef: -0.544, *P* = 0.040). Regarding marital status, we found that being married was significantly associated with reduction in frailty compared with unmarried older adults (Coef: -1.678, *P* < 0.001).

**Table 2:** Longitudinal associations between four types of social engagement and changes in frailty among Chinese older adults (*n* = 6562)

	Model 1		Model 2		Model 3		Model 4		Model 5		Model 6	
	Coef.	S.E.	Coef.	S.E.	Coef.	S.E.	Coef.	S.E.	Coef.	S.E.	Coef.	S.E.
Interaction with friends												
Not regularly	−0.569^***^	0.211									−0.568^***^	0.212
Almost every week	−1.186^***^	0.243									−1.152^***^	0.247
Almost daily	−1.358^***^	0.171									−1.309^***^	0.172
Hobby groups												
Not regularly			−0.832^***^	0.275							−0.694^**^	0.273
Almost every week			−0.829^***^	0.265							−0.544^**^	0.265
Almost daily			−1.429^***^	0.298							−1.189^***^	0.300
Sports groups												
Not regularly					−0.106	0.589					0.086	0.590
Almost every week					−0.338	0.515					−0.243	0.512
Almost daily					−1.075^***^	0.292					−0.945^***^	0.291
Voluntary work												
Not regularly							−0.120	0.226			0.065	0.227
Almost every week							−0.368	0.455			−0.047	0.456
Almost daily							−2.084^***^	0.604			−1.957^***^	0.598
Married							−1.644^***^	0.312			−1.678^***^	0.310

Note: All models were controlled for age, financial situation, currently working, smoking status, alcohol consumption, multi-morbidity, self-perceived health status, depression, and wave

Six individuals were excluded due to having missing covariate data, and seven individuals were not included due to more than 20% of deficits were missing

Standard errors all clustered at the individual level. *** *P* < 0.01, ** *P* < 0.05, * *P* < 0.1

Table 3 shows the results of interaction effects of social engagement and marital status on frailty. In models 7 to 10, we added the interaction term between each social engagement and marital status. We found that the interaction between often engaging in hobby groups and the married dummy was statistically significant (Coef: -1.280, *P* = 0.037). The interactions between marital status and the other three types of social engagement were not significantly at 5% level.

**Table 3:** The interaction effects of social engagement and marital status on frailty among Chinese older adults (*n* = 6562)

	Model 7		Model 8		Model 9		Model 10	
	Coef.	S.E.	Coef.	S.E.	Coef.	S.E.	Coef.	S.E.
Interaction with friends								
Not regularly × Married	−0.165	0.492						
Almost every week × Married	0.376	0.588						
Almost daily × Married	0.400	0.393						
Hobby groups								
Not regularly × Married			−0.230	0.701				
Almost every week × Married			−0.351	0.688				
Almost daily × Married			−1.280^**^	0.615				
Sports groups								
Not regularly × Married					−2.217	1.411		
Almost every week × Married					0.551	1.214		
Almost daily × Married					−0.041	0.719		
Voluntary work								
Not regularly × Married							0.501	0.565
Almost every week × Married							1.154	1.195
Almost daily × Married							−0.844	1.310

Note: All models were controlled for age, financial situation, currently working, smoking status, alcohol consumption, multi-morbidity, self-perceived health status, depression, and wave

Six individuals were excluded due to having missing covariate data, and seven individuals were not included due to more than 20% of deficits were missing

Standard errors all clustered at the individual level. *** *P* < 0.01, ** *P* < 0.05, * *P* < 0.1

### Sensitivity analyses

First, Supplementary Table 2 shows that frailty was not significantly associated with all four types of social engagement at the subsequent waves, suggesting that reverse causality appears not to be a concern. Second, we used an F-test to compare the POLS with the FE model. The *P*-value was statistically significant (*P* < 0.001), which indicated that the former might be biased. Then we used Hausman-test to assess whether random effect models were preferred over the FE models, and the test yielded *P*-value less than 0.001, indicating a preference over the FE models. Next, we investigated whether it was necessary to control for time-fixed effects in our models. After estimating the FE models with the wave dummy variables, we tested the null hypothesis that the coefficients of those variables were all equal to zero. Results (*P* < 0.001) indicated the necessity to control for time-fixed effects in the models. Finally, the results of multiple imputation did not differ much from the main results (see Supplementary Table 3).

## Discussion

The current study systematically examines the associations between four types of social engagement and physical frailty among a sample of individuals aged 60 and above from a longitudinal study in China, as well as whether these associations differs by marital status. Overall, after eliminating the time-invariant unobserved heterogeneity and any individual-level idiosyncratic disturbances over time, our study have offered longitudinal evidence that frequent interaction with friends, engaging in hobby groups, sports groups, and voluntary work are all associated with physical frailty reduction among older people, even after fully adjusting for potential confounding factors.

Few studies have examined the association between social engagement and frailty among older adults. One notable exception is Kwan et al. [23], which shows an association between social engagement and lower frailty using cross-sectional data. However, their research do not distinguish the specific types of social engagement. In this study, we examine each type of social engagement activity separately and find that more often volunteering is associated with frailty reduction. A review by Jenkinson et al. [45] suggests that volunteering benefits mental health and reduces mortality. Although volunteering has been shown to bring a wide range of beneficial effects for the volunteers, this is the first time that such health benefits have been demonstrated among the Chinese older adults. Further, the protective effect of volunteering on frailty is observed only in the almost daily group, suggesting that there may be a threshold effect. Proulx et al. [46] also suggests a threshold effect of volunteering on cognitive function. Similar to volunteering, our study shows often engaging in sports groups is associated with frailty reduction. Previous studies reports that older adults who engage in regular physical activities are associated with reduced FI [47]. Not only is this association replicated in the present study, but we find that engaging in sports groups remains robust after controlling for physical activities. In addition to the benefits of exercise itself, one of the explanations for this finding might be that engaging in sports groups may represent an opportunity for social interaction through groups’ activities or interaction with staff members. Furthermore, the advantages of engaging in sports clubs are providing specialist equipment and facilities, and the guidance or support from others [48].

Chinese older adults usually participate in hobby groups after retirement, such as playing Mahjong, chess, or card. Unlike engagement in voluntary work and sports groups, not only frequent engagement, but also not regular or some of the time engaging in hobby groups is associated with frailty reduction. Earlier studies [49] found that participation in hobby groups in late life is associated with better cognitive function and therefore related to cognitive aspects of FI. Similar to engaging in sports groups, engaging in hobby groups also establishes interpersonal social contact. Given the importance of interpersonal social contact, however, the most direct way to establish interpersonal relationship is interaction with friends, such as drop around. Our study shows those who often or occasionally interact with friends have lower FI. One cross-sectional study [50] suggests that interacting with friends one to four times per month is significantly associated with better control of diabetes.

Theoretically, social capital theory [51] provides a useful framework for understanding the potential protective effect of social engagement on frailty. According to this theory, social capital is defined as the “resources that are accessed by individuals as a result of their membership of a network or a group” [52], and social engagement is an important tool for older adults to obtain these social resources [19]. A sense of belonging, companionship, and social integration are positive dominant features which are brought by social engagement (through shared time and engagement in joint activities with others). These positive features enable people to obtain a variety of practical and health-improving resources, including but not limited to, self-efficacy, self-esteem, and social support, which will have a positive impact on health and well-being among older people. The concept of social frailty is similar to social capital, which is defined as the absence of social resources, social activities, and self-management abilities that are important for fulfilling one or more basic social needs during the life span [53]. According to Bunt’s social frailty concept framework [54], social engagement as one of the social behaviors can lead to the ‘fulfilment of basic social needs’, such as trusted relationships and experience of warm. This fulfilment of basic social needs in turn lends a positive impact to health and well-being. Berkman [55] developed a conceptual framework, known as the Berkman’s social relationship model, to further explain the relationship between social engagement and health. The model hypothesizes that social relationships can affect health through a series of causal processes that begin at the macrosocial level (upstream factors) to micro-psychobiological processes (downstream factors). Social engagement, as one of the “downstream factors”, can affect health through psychological and health behavioral mechanisms [56, 57]. Their interpretation of engagement focuses on a person’s ability to play a potential roles through social connections. Through some social activities, such as interaction with friends, engaging in group recreation, and volunteering, meaningful social roles are defined and reinforced, which in turn, provides a sense of value, belonging, and attachment. The consequences of social engagement in a meaningful social relationships or having a high degree of “connectedness” in a society eventually gives purpose to an individual’s life and that part of social engagement has been hypothesized to be a powerful predictor of health [58].

This study also shows that being married is significantly associated with the frailty reduction in older adults, which is consistent with previous studies [17, 28–30]. This finding offer support to the marriage protection theory that married adults have better physical health because of the increased social support and reduced incidence of risk behaviors among married individuals [59]. Marriage is a central resource for social support in later life due to the narrowing of social networks [60], especially when social engagement is limited. Marriage may promote health through various causal mechanisms, including gaining legal access to marital assets and resources, monitoring spouse’s health status and behaviors, and forming social bonds [61]. As such, the health effects of marriage cannot be ignored among older adults, and future interventions on frailty should pay more attention to unmarried individuals because they are more likely to suffer from frailty.

The results of interaction effect shows that marital status plays a moderating role in the relation between engaging in hobby groups and physical frailty reduction. Specifically, the association between frequent engaging in hobby groups and frailty reduction is particularly pronounced for participants who are not married. According to the model of marital resource [27], married individuals have greater access to social, psychological, and economic resources than unmarried people and in turn promote health and longevity. The lack of marriage makes social engagement an important way for unmarried older people to obtain resources, such as social support and social integration, which therefore makes them more likely to pay attention to and enjoy the process of social engagement. Thus, unmarried individuals may gain more benefits in psychological well-being and health-related behaviors through social engagement, and in turn reduce the physical frailty in old age. Taken together, our findings have important policy implications on the prevention of frailty. Future policy initiatives and interventions should pay particular attention to encourage social engagement among older adults, including interacting with friends, engaging in hobby groups, participating in sports groups, and volunteering regularly. The government should provide facilities for the social engagement among older adults, such as developing formal volunteer organizations, and building activity centers for older adults in the community. In particular, policymakers should encourage the unmarried older adults to engage in hobby groups, such as playing chess or Mahjong with others, because it may compensate health deficits for the unmarried status.

The present research contributes to existing literature by examining the longitudinal effects of social engagement on physical frailty among older people using longitudinal panel data from three waves of the CHARLS. The results suggests the concept of social engagement would have clinical relevance and provides new insights for physical frailty prevention. Methodologically, the associations may reflect confounding by unobserved characteristics or reverse causality from frailty to social engagement. The present study has two strengths in its analytical strategies. First, we use individual fixed-effects models to adjust for unobserved time-invariant confounders. Fixed-effects model exploit longitudinal nature of the data by assessing the association between changes in social engagement and changes in physical frailty within individuals, and thus controlling for unmeasured individual heterogeneity that may be correlated with both social engagement and frailty, such as socioeconomic status, personality traits, and intellectual ability [62]. This method can provide additional insights into the potential causal association by controlling for individual heterogeneity. Second, we conduct a test for the endogenous selection to limit the bias due to reverse causality.

This paper has several limitations. First, while we have found that frequent engaging in volunteer activity is associated with reductions in frailty, it might be possible to assess the exact threshold or dose-response relationship if more fine-grained data on volunteering hours are available. Unfortunately, our data do not allow for this, thus we leave this issue for future research. Second, although the FE models can eliminate the time-invariant unobserved heterogeneity and we have controlled for some time-variant confounding, there may still be residual time-varying confounding not controlled in our study. For example, older adults’ social support may be an important resource that can increase their health and thus protect against frailty reduction. We do not control for social support due to data limitation, which might have led to an over-estimation of our estimates. Finally, engaging in community-related organizations has not been included in our study because there is a limited number of older adults who engage in community-related organizations. In this case, the relationships between engaging in community-related organizations and frailty await future studies with a larger sample size. Our study may serve as a basis for future research evaluating the value of social engagement in designing targeted interventions of frailty. Prospective studies are needed to identify the causal relationship for social engagement and frailty, and further research is required to identify the specific mechanisms that explain the association between social engagement and frailty among older people.

## Conclusions

In conclusion, this large, longitudinal study suggests that frequent social engagement, including interaction with friends, engaging in hobby groups, sports groups, and volunteering, potentially results in lower physical frailty among older Chinese population. Furthermore, the association between engaging in hobby groups and frailty reduction is particularly pronounced in unmarried groups. These findings have important implications for public health policy and encourage the incorporation of a broad range of social engagement into the daily lives of older individuals to protect against frailty status among Chinese older people. Specially, encouraging unmarried older adults to engage in hobby groups, such as playing chess or Mahjong with others, may be an effective way to reduce frailty.

## Supplementary Information

**Additional file 1.**

## Data Availability

The datasets used in the current study are available in the CHARLS repository, http://charls.pku.edu.cn/pages/data/111/en.html.

## Abbreviations

FI: Frailty Index
CHARLS: the China Health and Retirement Longitudinal Study
HRS: the Health and Retirement Study
ELSA: the English Longitudinal Study of Aging
SHARE: the Survey of Health, Aging and Retirement in Europe
ADL: activities of daily living
IADL: instrumental activities of daily living
FE: fixed-effects
POLS: spooled ordinary least squares
RE: random-effects
