# Supplementary material for: Social engagement and physical frailty in later life: does marital status matter?
Source: BMC Geriatr. 2021 Apr 15;21:248. doi: 10.1186/s12877-021-02194-x (PMC8047563; doi:10.1186/s12877-021-02194-x)
Supplement: Supplementary file 1 — Additional file 1. [file 12877_2021_2194_MOESM1_ESM.docx]

**Appendix**

Social engagement and physical frailty in later life: does marital status matter?

**Author list:** Yi Wang, PhD^a^, Zhuo Chen, PhD^c, d^, and Chengchao Zhou, PhD^a, b,^ *

Yi Wang; Centre for Health Management and Policy Research, School of Public Health, Cheeloo College of Medicine, Shandong University, Jinan, 250012, China; wangyi031104@163.com

Zhuo Chen; College of Public Health, University of Georgia, Athens, Georgia, 30602, United States; School of Economics, University of Nottingham Ningbo China, Ningbo, 315100, China; [zchen1@uga.edu](mailto:zchen1@uga.edu)

Chengchao Zhou; Centre for Health Management and Policy Research, School of Public Health, Cheeloo College of Medicine, Shandong University, Jinan, 250012, China; NHC Key Laboratory of Health Economics and Policy Research, Shandong University; [zhouchengchao@sdu.edu.cn](mailto:zhouchengchao@sdu.edu.cn)

**Corresponding Author:* Chengchao Zhou (Prof.), Centre for Health Management and Policy Research, School of Public Health, Cheeloo College of Medicine, Shandong University; NHC Key Laboratory of Health Economics and Policy Research, Shandong University; 44 Wen-hua-xi Road, Jinan, Shandong, 250012, China

Tel: (+86) 531 8838 1567 Fax: (+86) 531 8838 2553

**Contents:**

- **Supplementary Table 1.** List of items included in the Frailty Index
- **Supplementary Table 2.** Test for the endogenous selection (reversed causality)
- **Supplementary Table 3.** Fixed-effects models predicting the frailty with social engagement and marital status using multiple imputation techniques

**Supplementary Table 1.** List of items included in the Frailty Index

| No. | Variables | Values |
| --- | --- | --- |
| 1 | Self-reported health | very good = 0; good = 0.25; fair = 0.5; poor = 0.75; very poor = 1 |
| 2 | Some difficulty with dressing | yes = 1; no = 0 |
| 3 | Some difficulty with bathing | yes = 1; no = 0 |
| 4 | Some difficulty with eating | yes = 1; no = 0 |
| 5 | Some difficulty with get/in bed | yes = 1; no = 0 |
| 6 | Some difficulty with using the toilet | yes = 1; no = 0 |
| 7 | Some difficulty with controlling urination and defecation | yes = 1; no = 0 |
| 8 | Some difficulty with managing money | yes = 1; no = 0 |
| 9 | Some difficulty with take medications | yes = 1; no = 0 |
| 10 | Some difficulty with shop for grocery | yes = 1; no = 0 |
| 11 | Some difficulty with prepare hot meal | yes = 1; no = 0 |
| 12 | Some difficulty with cleaning house | yes = 1; no = 0 |
| 13 | Mathematical performance | 5 correct responses = 0; 4 correct = 0.2; 3 correct = 0.4; 2 correct = 0.6, 1 correct = 0.8; 0 correct = 1 |
| 14 | Orientation | 4 correct responses = 0; 3 correct = 0.25; 2 correct = 0.5; 1 correct = 0.75; 0 correct = 1 |
| 15 | Draw assign picture | yes = 0; no = 1 |
| 16 | High blood pressure | yes = 1; no = 0 |
| 17 | Diabetes | yes = 1; no = 0 |
| 18 | Cancer | yes = 1; no = 0 |
| 19 | Lung disease | yes = 1; no = 0 |
| 20 | Heart problem | yes = 1; no = 0 |
| 21 | Stroke | yes = 1; no = 0 |
| 22 | Psychiatric problem | yes = 1; no = 0 |
| 23 | Arthritis | yes = 1; no = 0 |
| 24 | Dyslipidemia | yes = 1; no = 0 |
| 25 | Liver disease | yes = 1; no = 0 |
| 26 | Kidney disease | yes = 1; no = 0 |
| 27 | Stomach/digestive disease | yes = 1; no = 0 |
| 28 | Asthma | yes = 1; no = 0 |
| 29 | Sleep was restless | yes = 1; no = 0 |
| 30 | Felt lonely | yes = 1; no = 0 |
| 31 | Could not get going | yes = 1; no = 0 |
| 32 | Feel hopeful about the future | yes = 0; no = 1 |

**Supplementary Table 2.** Test for the endogenous selection (reversed causality)

|  | Interaction with friends_2015 | | Hobby groups_2015 | | Sports groups_2015 | | Volunteer activities_2015 | |
| --- | --- | --- | --- | --- | --- | --- | --- | --- |
|  | Coef. | *P*-value | Coef. | *P*-value | Coef. | *P*-value | Coef. | *P*-value |
| FI_2013 | 0.002 | 0.266 | 0.001 | 0.742 | -0.001 | 0.647 | 0.001 | 0.123 |
| FI_2011+FI_2013 | YES |  | YES |  | YES |  | YES |  |

**Supplementary Table 3.** Fixed-effects models predicting the frailty with social engagement and marital status using multiple imputation techniques (n = 6,575)

|  | Model 1 |  | Model 2 |  | Model 3 |  | Model 4 |  | Model 5 |  |
| --- | --- | --- | --- | --- | --- | --- | --- | --- | --- | --- |
|  | Coef. | S.E. | Coef. | S.E. | Coef. | S.E. | Coef. | S.E. | Coef. | S.E. |
| Interaction with friends |  |  |  |  |  |  |  |  |  |  |
| Not regularly | -0.536^**^ | 0.212 | -0.367 | 0.435 | -0.544^**^ | 0.212 | -0.531^**^ | 0.212 | -0.539^**^ | 0.212 |
| Almost every week | -0.984^***^ | 0.248 | -1.230^**^ | 0.537 | -0.988^***^ | 0.248 | -0.987^***^ | 0.248 | -0.983^***^ | 0.249 |
| Almost daily | -1.162^***^ | 0.174 | -1.380^***^ | 0.350 | -1.165^***^ | 0.174 | -1.159^***^ | 0.174 | -1.160^***^ | 0.174 |
| Hobby groups |  |  |  |  |  |  |  |  |  |  |
| Not regularly | -0.713^**^ | 0.277 | -0.713^**^ | 0.277 | -0.575 | 0.678 | -0.723^***^ | 0.277 | -0.705^**^ | 0.277 |
| Almost every week | -0.567^**^ | 0.270 | -0.574^**^ | 0.270 | -0.276 | 0.659 | -0.574^**^ | 0.270 | -0.566^**^ | 0.270 |
| Almost daily | -1.188^***^ | 0.302 | -1.193^***^ | 0.302 | -0.035 | 0.569 | -1.201^***^ | 0.302 | -1.188^***^ | 0.302 |
| Sports groups |  |  |  |  |  |  |  |  |  |  |
| Not regularly | 0.043 | 0.587 | 0.042 | 0.587 | 0.033 | 0.587 | 1.929 | 1.224 | 0.029 | 0.588 |
| Almost every week | -0.260 | 0.514 | -0.262 | 0.515 | -0.256 | 0.515 | -0.580 | 1.038 | -0.269 | 0.514 |
| Almost daily | -0.892^***^ | 0.294 | -0.893^***^ | 0.294 | -0.867^***^ | 0.294 | -0.878 | 0.665 | -0.889^***^ | 0.294 |
| Voluntary work |  |  |  |  |  |  |  |  |  |  |
| Not regularly | 0.193 | 0.227 | 0.194 | 0.227 | 0.194 | 0.227 | 0.203 | 0.227 | -0.144 | 0.520 |
| Almost every week | 0.003 | 0.462 | 0.014 | 0.463 | -0.001 | 0.463 | 0.005 | 0.462 | -1.023 | 1.129 |
| Almost daily | -1.835^***^ | 0.591 | -1.818^***^ | 0.591 | -1.826^***^ | 0.592 | -1.836^***^ | 0.591 | -1.374 | 1.083 |
| Married | -1.714^***^ | 0.309 | -1.764 | 0.330 | -1.604^***^ | 0.325 | -1.699^***^ | 0.313 | -1.757^***^ | 0.315 |
| Interaction with friends |  |  |  |  |  |  |  |  |  |  |
| Not regularly × Married |  |  | -0.223 | 0.495 |  |  |  |  |  |  |
| Almost every week × Married |  |  | 0.339 | 0.584 |  |  |  |  |  |  |
| Almost daily × Married |  |  | 0.311 | 0.395 |  |  |  |  |  |  |
| Hobby groups |  |  |  |  |  |  |  |  |  |  |
| Not regularly × Married |  |  |  |  | -0.181 | 0.722 |  |  |  |  |
| Almost every week × Married |  |  |  |  | -0.383 | 0.706 |  |  |  |  |
| Almost daily × Married |  |  |  |  | -1.500^**^ | 0.630 |  |  |  |  |
| Sports groups |  |  |  |  |  |  |  |  |  |  |
| Not regularly × Married |  |  |  |  |  |  | -2.409 | 1.391 |  |  |
| Almost every week × Married |  |  |  |  |  |  | 0.403 | 1.192 |  |  |
| Almost daily × Married |  |  |  |  |  |  | -0.016 | 0.728 |  |  |
| Voluntary work |  |  |  |  |  |  |  |  |  |  |
| Not regularly × Married |  |  |  |  |  |  |  |  | 0.436 | 0.567 |
| Almost every week × Married |  |  |  |  |  |  |  |  | 1.357 | 1.222 |
| Almost daily × Married |  |  |  |  |  |  |  |  | -0.689 | 1.290 |

Note: All models were controlled for age, financial situation, currently working, smoking status, alcohol consumption, multi-morbidity, self-perceived health status, depression, physical activity, and wave.

Standard errors all clustered at the individual level. *** *P* < 0.01, ** *P* < 0.05, * *P* < 0.1.
